# Supplementary material for: Splice-Junction-Based Mapping of Alternative Isoforms in the Human Proteome
Source: Cell Rep. Author manuscript; Available in PMC 2020 Jan 15. (PMC6961840; doi:10.1016/j.celrep.2019.11.026)
Supplement: 2 [file NIHMS1546469-supplement-2.zip › DF1/PXD009737/TestisGluC/TestisGluC_12_WNK1_GPVLATSSGAGVFKMGRFQPVSMAAPTAIT.pdf]

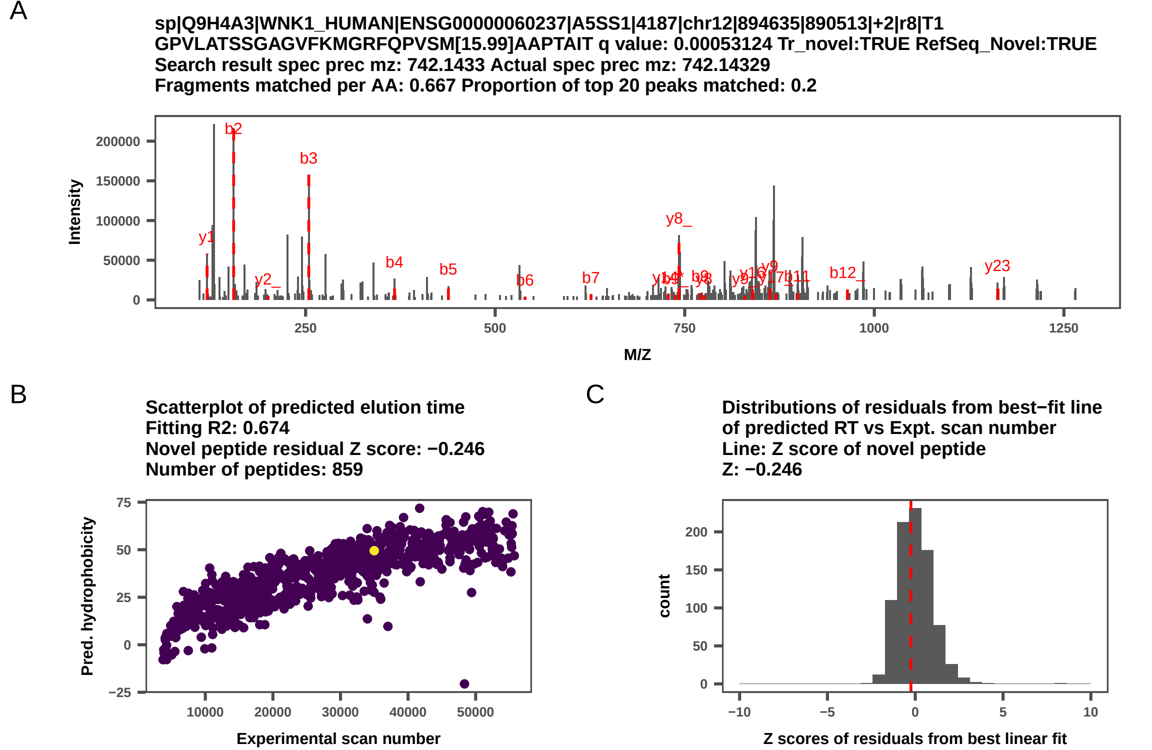
A
sp|Q9H4A3|WNK1_HUMAN|ENSG00000060237|A5SS1|4187|chr12|894635|890513|+2|r8|T1
GPVLATSSGAGVFKMGRFQPVSM[15.99]AAPTAIT q value: 0.00053124 Tr_novel:TRUE RefSeq_Novel:TRUE
Search result spec prec mz: 742.1433 Actual spec prec mz: 742.14329
Fragments matched per AA: 0.667 Proportion of top 20 peaks matched: 0.2
Intensity
200000
150000
100000
50000
0
b2
b3
y1
y2_
b4
b5
b6
b7
y8_
b12_
y23
250
500
750
1000
1250
M/Z
B
Scatterplot of predicted elution time
Fitting R2: 0.674
Novel peptide residual Z score: −0.246
Number of peptides: 859
Pred. hydrophobicity
75
50
25
0
−25
10000
20000
30000
40000
50000
Experimental scan number
C
Distributions of residuals from best−fit line
of predicted RT vs Expt. scan number
Line: Z score of novel peptide
Z: −0.246
count
200
150
100
50
0
−10
−5
0
5
10
Z scores of residuals from best linear fit
